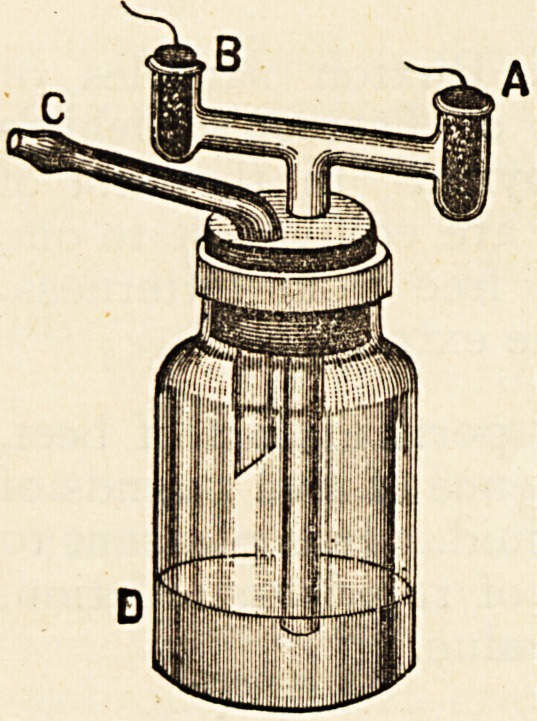# Notes on Preparations for the Sick

**Published:** 1888-09

**Authors:** 


### Notes on Preparations for the Sick.

**Chloride of Ammonium Inhaler. Drugs in Capsules.**—T. Buxton, Bristol.

The **Inhaler** is one of the most simple, and at the same time most efficient, of the numerous devices for supplying chloride of ammonium. It yields a plentiful supply of the gas, which is well washed, and does not pass through any Indiarubber tubes. It cannot well fail in action, and will be always ready whenever the plugs are freshly charged with ammonia and hydrochloric acid. The simplicity of the apparatus will be seen by the drawing and the directions for charging and using. First. Fill the bottle D one-third full with water. Second. Dip the porous plug A into the solution of ammonia, drain off excess of ammonia and replace it in the chamber A. Third. Dip the porous plug B into the hydrochloric acid, drain off excess of acid and replace it in the chamber B. *The Inhaler is now charged.* Place the mouthpiece C between the lips, and inhale by drawing the vapour into the mouth and throat, then exhale by breathing naturally through the nostrils. This should be repeated for about five minutes at a time and at least three times a day. N.B. Be careful never to blow into the Inhaler, and see that the plugs fit loosely.

The fumes of chloride of ammonium will be found useful in many acute and chronic affections of the throat and air-passages.

Each of the **Creasote Capsules** contains five minims of the drug. The value of creasote appears to depend to a great extent on the dose in which it is given. The powerful taste prevents its full administration in the fluid form, even in combination with tinctures, glycerine, or cod-liver oil. The capsule is one method by which full doses may be given: one or more taken after food are slowly dissolved, the contents mix with the food, and cause no after-taste or interference with the digestion.

The **Creasote and Iodoform Capsules** each contain five minims of creasote with one grain of iodoform suspended in it. They are a convenient form in which two nauseous, but useful, drugs may be easily administered without risk of gastric irritation. Drugs of this kind, which have to be continued for months or perhaps years, are better given in the capsule-form, as they are less likely to weary and disgust the patient by either their taste or odour.

In the **Cascara Sagrada Capsules** the nauseous taste of a superlatively nauseous drug is effectually covered by enclosing an inspissated fluid extract in gelatine. Each capsule represents thirty drops of the fluid extract. They are efficient in action, and are altogether a success.

We have also received from Mr. Buxton samples of **Liq. Cascaræ Sagradæ Dulcis** (Evans), of **Pearson's Liebig's Beef Wine,** and of **Effervescing Antipyrin.** In the first of these preparations two fluid drachms are equivalent to one drachm of the bark. It is absolutely free from bitterness, but retains the full aperient value of the extract.

The **Beef Wine** is a combination of port, extract of beef, quinine, and extract of malt. The essence of five pounds of beef is contained in each quart of the fluid. It is pleasant to the palate, its ingredients are capable of rapid assimilation, and it should be of much therapeutic value.

The analgesic and hæmostatic properties of **Antipyrin,** in combination with its antipyretic powers, give it a very wide range of utility; and the effervescing form is one of the most agreeable modes for its administration. Each drachm contains five grains of the drug, and is sufficient for many cases of nervous headache, and slight pains or pyrexia: in more severe cases this dose may be doubled, and may be given every hour till the pain is relieved or the temperature has fallen. In the pyrexia of phthisis we have found it materially add to the comfort of the patient, and in many nerve cases it acts better than the bromides.

**Sodium Silico-fluoride.**—Winser & Co., Manchester.

This, which is also known as Salufer, is a powerful antiseptic and disinfectant, non-volatile, non-poisonous, free from smell, and does not stain. Water will not dissolve more than 1 ounce to the gallon; but a $\frac{1}{4}$-ounce per gallon is sufficient for disinfecting purposes. Mr. William Thomson of Manchester reports that of the compounds of fluorine this is the most powerful, and that a saturated solution of this salt preserves chopped meat better than 1 in 500 of mercurial bichloride. The salt has been used by Mr. Mayo Robson for general surgical purposes, more especially when the mercurial bichloride is contra-indicated; and the results given were satisfactory. The powder was found to be a strong irritant; but a solution of one grain to the ounce was not so. Hydrofluoric acid has recently been credited with success in the treatment of phthisis: possibly Salufer may prove to be still more useful.

---

**Dr. Angell's Milk Food.**—R. Sumner & Co., Liverpool.

This **Food** is the most improved form of the infants' food originally devised by Liebig: "it is cooked and pancreatised." Being easy of digestion, it must be of service to the dyspeptic, who cannot well have too much variety in this kind of food, none of which he likes, although he cannot digest anything stronger.

We have submitted this food to microscopic examination, and find that the starch-granules show no indications of the action of heat. It appears to us that it must be a mistake to speak of unbroken starch-granules as cooked; certainly they require further cooking before they can become fitted for human aliment, inasmuch as it is generally admitted that unbroken starch-granules pass unchanged through the alimentary canal of man.

---

**Scott's Emulsion of Pure Cod Liver Oil with Hypophosphites of Lime and Soda.**—Scott & Bowne, London.

This now well known **Emulsion** contains 50 per cent. of the oil with six grains of hypophosphite of lime and three grains of the soda salt in each fluid ounce. It is a permanent emulsion, is not liable to decompose or grow rancid; it has a large percentage of glycerine, giving it a sweet taste, is palatable, easily digested, and is shown by experience to be effective in counteracting many states of defective nutrition.

It is one of the best emulsions we have given, and will not disappoint those who use it.

---

**Kola Chocolate.**—Thomas Christy & Co., London.

This is prepared from the powdered nut of the Sterculia acuminata: it contains a large percentage of caffeine, and hence are probably due the marvellous sustaining powers credited to the fresh and dry nut by the natives on the West Coast of Africa and in the West Indies. The bitterness and astringency of the nut impart tone to the digestive organs, and have a beneficial action in diarrhœa and dysentery.

Both as a fluid beverage, and as a food product, the **Kola Chocolate** forms an agreeable preparation, well borne by the dyspeptic, and of use in the treatment of neurasthenia, dipsomania, insomnia, and other unruly disorders of the nervous system.

The Kola Tablets, convenient for those who cannot prepare the Chocolate twice a day, have been found useful to those engaged in mental or bodily fatigue: in long journeys, when meals are necessarily irregular, their sustaining properties will be much appreciated.

---

**Vichy "Pastilles Digestives."**—Ingram & Royle, London.

These **Pastilles Digestives** of the Vichy Thermal Establishment are prepared with the natural salts extracted from the Vichy waters by the Compagnie Fermière. They are flavoured with peppermint, lemon, vanilla, rose, tolu, aniseed, or orange flower: they may also be obtained unflavoured. They have obtained a considerable reputation for their convenience and utility in the treatment of some of the varieties of dyspepsia. Where the sugar is not a disadvantage, they are a pleasant and efficient method of administration of small doses of alkali in the lozenge form.

**Unsweetened Condensed Milk.**—The First Swiss Condensed Milk Co., London.

This preparation is simply pure, unskimmed, Alpine milk of good quality, without the addition of sugar or any other preservative whatever. It is uniform in quality, will keep well in any climate, and on dilution forms a good ordinary milk, with the normal proportion of nitrogenous and other constituents. Sufficient heat is used in the process of condensation to destroy all putrefactive germs: it follows that pathogenic germs must be destroyed likewise, if any were present. This milk is therefore an effectual safeguard, to those who use no other, from such contagious diseases as scarlet fever, typhoid, tuberculosis, &c., which have frequently been traced to the milk supply. The purest milk, further purified by heat, and preserved in hermetically sealed tins, cannot carry infection, and is always within reach by dilution of this manufacture of the Alpine Milk Exporting Company at Romanshorn.

---

**Condal Water.**—23 Lime Street, London.

This is a natural aperient water from the springs at Rubinat, in Spain, bottled at the source, and not manufactured in any way. It is not a bitter water, and it does not deteriorate when uncorked. We have found it to be a most efficient aperient, and the dose is easily graduated in accordance with the effect desired.

## Figures and Tables

**Figure f1:**